# Risk of lymph node metastases in multifocal papillary thyroid cancer associated with Hashimoto's thyroiditis

**DOI:** 10.1007/s00423-013-1158-2

**Published:** 2014-01-10

**Authors:** Aleksander Konturek, Marcin Barczyński, Wojciech Nowak, Wojciech Wierzchowski

**Affiliations:** 1 Department of Endocrine Surgery, Third Chair of General Surgery, Jagiellonian University Medical College, 37 Prądnicka Street, Kraków, 31-202 Poland; 2 Department of Pathology, Jagiellonian University Medical College, 16 Grzegórzecka Street, Kraków, 31-531 Poland

**Keywords:** Hashimoto’s thyroiditis, Multifocal papillary thyroid cancer, Benign thyroid disease

## Abstract

**Aims:**

The aim of this study was to evaluate the risk factors of lymph nodes metastases (LNM) in patients with papillary thyroid cancer (PTC) and coexisting Hashimoto’s thyroiditis (HT).

**Patients and methods:**

This was a retrospective cohort study of patients with PTC and HT who had undergone total thyroidectomy (TT) with central neck dissection (CND) over an 11-year period (between 2002 and 2012). Pathological reports of all eligible patients were reviewed. Multivariable analysis was performed to identify risk factors of LNM.

**Results:**

During the study period, PTC was diagnosed in 130 patients with HT who had undergone TT with CND (F/M ratio = 110:20; median age, 52.4 ± 12.7 years). Multifocal lesions were observed in 28 (21.5 %) patients. LNM were identified in 25 of 28 (89.3 %) patients with multifocal PTC and HT versus 69 of 102 (67.5 %) patients with a solitary focus of PTC and HT (*p* = 0.023). In multivariable analysis, multifocal disease was identified as an independent risk factor for LNM (odds ratio, 3.99; 95 % confidence interval, 1.12 to 14.15; *p* = 0.033).

**Conclusions:**

Multifocal PTC in patients with HT is associated with an increased risk of LNM. Nevertheless, the clinical importance of this finding needs to be validated in well-designed prospective studies.

## Introduction

Papillary thyroid cancer (PTC) is among the most common forms of thyroid carcinomas, and its incidence has been rapidly increasing over the last years, coexisting with autoimmune Hashimoto’s thyroiditis-type thyroid diseases. Numerous data from the literature on the subject and multi-center meta-analyses demonstrate that PTC coexists almost three times more often with Hashimoto’s thyroiditis, and its concomitant occurrence reported in various publications ranges between 0.5 and 58 %. There are no unambiguous premises for stating whether an autoimmune disease predisposes to cancer development or else whether, in the course of cell transformation in the carcinogenesis process, there occurs a change in the autoimmune response that favors the development of such a disease. It is much more common in younger women with small primary foci or with multifocal disease and a statistically better prognosis as compared to the remaining patients [1–9].

Although the pathogenesis still remains highly controversial, the immune background of the disease, which involves activation of T-helper and T-cytotoxic lymphocytes and overproduction of specific antibodies (anti-TPO; anti-TG) favors papillary cell damage, leading in consequence to hypothyroidism. The relation between Hashimoto’s thyroiditis and papillary thyroid carcinoma, described for the first time by Daley in 1955, still continues to be a subject of discussion and attempts at explaining the phenomenon [10, 11].

On the one hand, it is suggested that under specific conditions, development of Hashimoto’s disease is a response to cancer cells appearing within the thyroid gland, while on the other, data are reported in support of a thesis that the observed morphological lesions in the thyroid result from immune disturbances leading to imbalance between natural death of a cell in keeping with the mechanism of apoptosis and uncontrolled proliferation of the cell [12, 13].

The objective of the report that is based on material collected at the department is to present the occurrence of lymph node metastases to the central compartment of the neck that coexist with the multifocal form of papillary thyroid cancer concomitant with Hashimoto’s thyroiditis. In the opinion of the authors, an early detection of such lesions, selection of an appropriate strategy of surgical management performed in selected referral centers, targeted adjuvant therapy, and treatment monitoring at the same time meeting the safety requirement of protecting the laryngeal nerves and preventing hypoparathyroidism may play a significant role in improving late therapeutic results [14].

## Materials and methods

Between January 2002 and December 2012, of 9,145 patients treated surgically in a single referral university center due to various thyroid diseases, a subgroup of 548 patients diagnosed with Hashimoto’s thyroiditis and coexistent papillary thyroid cancer was selected. The mean age of the patients was 52.4 ± 12.7 years and the female to male ratio was 464:84, respectively. Table 1 presents the demographic data of the study group. The analyzed group of patients with Hashimoto’s thyroiditis included all individuals meeting the following criteria: (1) high anti-thyroid peroxidase antibodies titers (anti-TPO), (2) lesions visualized by ultrasonography showing a hypoechoic or hyperechoic nodular pattern at least 5 mm in diameter, identification of a perinodular hypoechogenic or hyperechogenic halo and presence of an anechoic lesion with a reinforced posterior wall, (3) histology: presence of a diffuse lymphocytic infiltrate in the thyroid parenchyma and stroma with reaction foci and lymphatic follicles, presence of small follicles with a decreased colloid volume, foci of fibrosis and oxyphilic cytoplasm-containing cells. A repeated analysis of FNA results based on the 2009 Bethesda classification showed the descriptions of findings to most commonly include lesions that would be presently classified as belonging to grades III and IV.

**Table 1:** Clinical and pathological factors characteristics of 9,145 patients undergoing surgery in the years 2002–2012

	HT (*n* = 548)	Non-HT (*n* = 8,597)	*P*
Age, mean ± SD (years)	52.4 ± 12.7	53.3 ± 15.2	0.739
Sex ratio (M/F), no.	84 : 464	812 : 7785	0.484
Preoperative thyroid volume (by ultrasound), mean ± SD (ml)	22.1 ± 18.2	96.2 ± 40.4	<0.001
Anti-TPO (IU/ml)	428.6 ± 627.8	12.3 ± 7.8	<0.001
Preoperative diagnosis of benign thyroid disease, no. (%)	501 (91.4)	8,279 (96.3)	<0.001
Positive FNAB for PTC, no. (%)	47 (8.6)	318 (3.7)	<0.001

*HT* Hashimoto thyroiditis, *FNAB* fine needle aspiration biopsy, *PTC* papillary thyroid cancer

The exclusion criteria included patients positive for thyroid-stimulating hormone receptor antibodies (TRAb), Graves’ disease in medical history, and absence of clinical, ultrasound, and morphological signs of Hashimoto’s thyroiditis.

In the non-HT group, indications for surgical treatment included suspicious lesions detected by fine needle aspiration biopsy (FNAB; Bethesda grades III and IV: III—atypia of undetermined significance or follicular lesion of undetermined significance; IV—follicular neoplasm or suspicious for follicular neoplasm (Hurthle cell)), signs of compression of the trachea and surrounding tissues, multinodular toxic goiter, and Graves’ disease [15–17].

In keeping with the surgical management tactics adopted by the center represented by the authors, the scope of the primary operations performed in the presented group of patients with nodular variant of Hashimoto’s thyroiditis included total thyroidectomy combined with resection of the lymphatic system situated in the central compartment of the neck in 93 % of the patients; in the remaining individuals, subtotal bilateral lobectomies were performed. Final histology confirming a neoplastic pattern was an indication for total resection of residual thyroid tissue [18, 19].

All the procedures were performed by an experienced team of surgeons. In all the total thyroidectomy cases, the operation included extracapsular bilateral lobectomy combined with resection of the Delphic pretracheal and paratracheal lymph nodes (according to the American Thyroid Association classification) [20, 21].

In the majority of the patients with positive preoperative FNA biopsy results indicative of papillary thyroid cancer, identification of laryngeal nerves was achieved by intraoperative nerve monitoring: NEUROSIGN 100 System (Inomed, Emmendingen, Germany), which was used between the years 2004 and 2008, or NIM-Response 3.0 System (Medtronic Xomed, Jacksonville, FL), which was used between the years 2009 and 2012. In each instance, attempts were made to identify the four parathyroids and leave them in situ, exercising caution while dissecting the thyroid. In each case of parathyroid resection or doubts as to the appropriate parathyroids vasculature, the gland was reimplanted to the sternocleidomastoid muscle on the side of the original parathyroid location. The assessment of cancer stage was based on the seventh edition of the TNM classification of 2010. The histology results based on the repeated analysis of histological records stored in the Institutional Register of Thyroid Surgery were classified based on the above-presented assumptions.

Examinations performed in all the patients qualified for surgical treatment included thyroid ultrasonography and determinations of fT4, fT3, thyroid-stimulating hormone (TSH), and anti-TPO and anti-TG antibodies. In all the patients presenting with clinical and biochemical signs of hypothyreosis, levothyroxine substitution was initiated preoperatively. In the analyzed group, the mean follow-up was 5.1 ± 2.4 years. In each instance, in the first postoperative day, indirect laryngoscopy was performed and calcium levels were determined; hypocalcemia was diagnosed at total calcium levels below 2.0 mmol/l.

In all the patients after thyroid parenchyma resection and following establishing the diagnosis of differentiated thyroid cancer, a uniform therapeutic management strategy was adopted based on supplementation therapy with radioiodine in case of tumors with the diameter of >10 mm, administration of levothyroxine (at a dose providing a suppression level for TSH values 0.1–0.3 mU/L in the low-risk group and below 0.1 mU/L in the high-risk group), strict periodic determinations of TSH, TG, and anti-TG levels. The above management protocol is based on the recommendations of the Polish Group of Endocrine Tumors of 2010 [22].

In the statistical analysis, the comparison of variables with normal distribution was based on the Student’s *t* test, while the remaining variables were analyzed employing the *χ* ^2^ test. In the analyzed group, the “exposure” to the presented factors was determined based on the odds ratio. The multivariate analysis of lymph node metastases risk factors in case of coexisting HT-PTC was based on gender, age, smoking, central neck dissection, tumor size, multifocal disease, extension through the thyroid capsule, positive lymph nodes, and RAI treatment. Variables achieving statistical significance at the *p* ≤ 0.10 level in the univariable analysis were included in the multivariable analysis. A backward variable selection procedure with the cut-off at *P* < 0.050 was used to identify independent predictors of central lymph nodes involvement in HT.

All the data were collected prospectively and stored in a computer-based institutional register of thyroid surgery and analyzed retrospectively by a statistician. The statistical analyses were performed with Statistica 10 for Windows (StatSoft, Krakow, Poland).

## Results

The analyzed group of 548 patients with Hashimoto’s thyroiditis included 130 (23.5 %) cases of papillary thyroid carcinoma (HT-PTC) as compared to 643 (7.5 %) cancer patients in the group of 8,597 patients (non-HT-PTC) without diagnosed autoimmune disease. The values were statistically significant (*p* < 0.001). In the two analyzed groups, the ratio of males to females was different (HT-PTC group = 1:6; non-HT-PTC group = 1:10 The mean age and number of the surgical patients in the two analyzed groups were similar, showing the following respective values: (HT-PT group, 52.4 ± 12.7 versus non-HT-PTC group, 53.3 ± 15.2) and (<45 years: HT-PTC group: 52 = 40.0 % versus non-HT-PTC group: 278 = 43.2 %; ≥45 years: HT-PTC group: 78 = 60.0 % versus non-HT-PTC group: 365 = 56.8 %).

The size of goiter in the two analyzed groups assessed based on preoperative ultrasound volume measurements was markedly different (HT-PTC group = 22.1 ± 18.2; non-HT-PTC group = 96.2 ± 40.4). The difference was statistically significant (*p* < 0.001). Also, the difference in preoperative anti-TPO titers was statistically significant (*p* < 0.001) (HT-PTC group = 428.6 ± 627.8; non-HT-PTC group = 12.3 ± 7.8). Preoperatively, a positive result of fine needle aspiration biopsy (Bethesda grades V and VI) was noted in 47 (36.2 %) of 130 patients with HT-PTC. In the remaining cases, in the biopsy smears (FNAB—Bethesda grades III and IV), oxyphilic cells predominated along with lymphocytes and macrophages (Table 1).

According to the American Joint Committee on Cancer/Union Internationale Contre le Cancer, stage pT_1a_ papillary thyroid cancer was diagnosed in 80 (61.7 %) patients with Hashimoto’s thyroiditis and was predominant as compared to the remaining analyzed group (pT_1a_: HT-PTC group: 80 = 14.6 % versus non-HT-PTC group: 365 = 2.24 %; *p* < 0.001).

Multifocal cancer was noted in 28 (pTm = 21.5 %; respectively: pT1m = 25 (19.2 %); pT2m = 1 (0.8 %); pT3m = 2 (1.5 %)) patients with Hashimoto’s thyroiditis (HT-PTC group: 28 = 5.1 % versus non-HT-PTC group: 121 = 1.4 %). The multifocal form was defined as the presence of two or more neoplastic foci in one or both thyroid lobes. The diameter of the largest single neoplastic focus in the two analyzed groups was similar (HT-PT group: 8.7 ± 5.9 versus non-HT-PTC group: 9.4 ± 6.9); nevertheless, in a considerable number of patients (pT1m: HT-PTC: 28 = 21.5 %), the lesions were 10–12 and 2–3 mm in size, respectively, and were located bilaterally. The size of primary focus: tumor size (millimeters) <10 mm = 80 patients; ≥10 mm = 50; with infiltration of the thyroid capsule in 16 patients (in the group of patients with tumors >20 mm). Thyroid capsule infiltration predominated in unifocal forms. In multifocal tumors, the lesions usually involved both thyroid lobes and were situated in the central part of the gland or in the vicinity of the inferior poles, surrounded by lymphocyte infiltrations and the remaining thyroid tissue. Such a location of lesions in a great measure reflects our knowledge on the lymphatic outflow routes from the thyroid to the central lymph nodes, at the same time explaining the number of the involved central compartment cervical lymph nodes in the presently analyzed material.

**Fig. 1:**
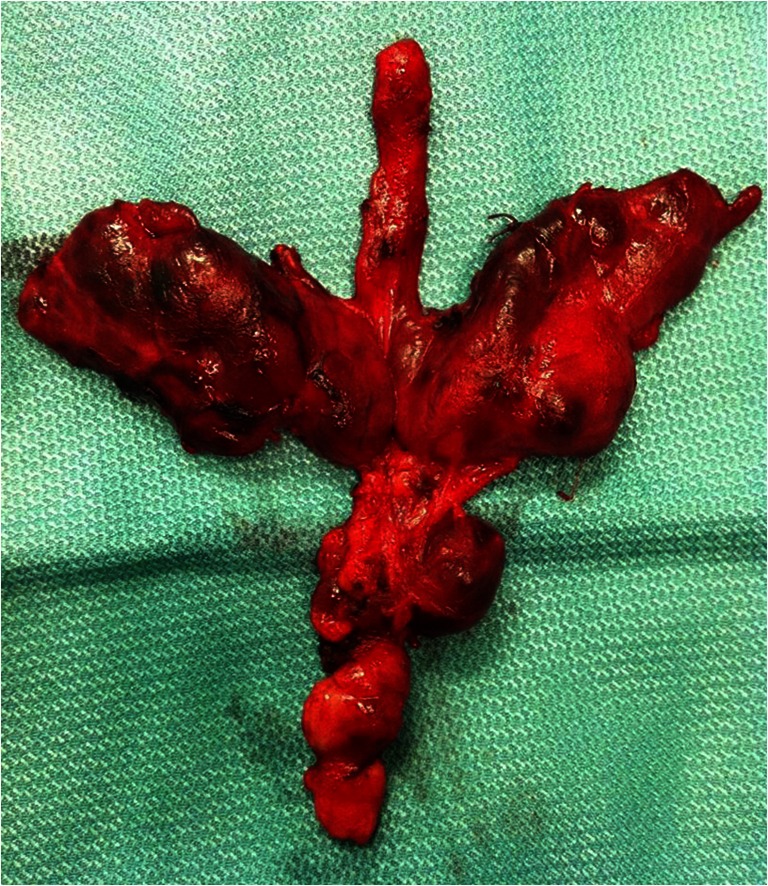
Specimen of en-block resected thyroid gland with level VI lymph nodes

A statistically significant difference was seen in lymph node metastases to the central compartment of the neck (compartment VI). LNM were identified in 25 of 28 (89.3 %) patients with multifocal PTC and HT versus 69 of 102 (67.5 %) patients with a solitary focus in the HT-PTC group (*p* = 0.023). In patients with HT, preparations originating from the compartment VI demonstrated respectively a higher number of resected and a higher number of metastatically involved lymph nodes as compared to the remaining group of non-HT patients (HT-PTC group: 13.8 ± 3.7 versus non-HT-PTC group: 6.5 ± 2.7 and HT-PTC group 6.4 ± 2.7 versus non-HT-PTC group: 2.1 ± 1.0) (Fig. 1; Table 2).

**Table 2:** Final histopathology in 773 patients with PTC involved in this study in relation to the Hashimoto thyroiditis

	PTC with HT (*n* = 130)	PTC in non-HT (*n* = 643)	*P*
Sex ratio (M/F)	20:110	69:574	0.129
Age at diagnosis (years)			
<45	52 (40.0)	278 (43.2)	0.496
≥45	78 (60.0)	365 (56.8)	0.496
pT1a	80 (61.5)	391 (61.7)	0.569
pT1b	22 (14.6)	57 (8.9)	0.006
pT2	12 (9.2)	78 (12.1)	0.295
pT3	16 (12.6)	108 (16.9)	0.168
Multifocal lesions	28 (21.5)	121 (18.8)	0.469
pN1a	94 (72.3)	221 (34.9)	<0.001
pT1aN1a	47 (36.1)	37 (5.8)	<0.001
pT1bN1a	18 (13.8)	51 (7.9)	0.031
pT2N1a	2 (1.5)	15 (2.3)	0.573
pT3N1a	11 (8.5)	87 (13.7)	0.094
Multifocal lesions and N_1a_	25 (19.2)	51 (7.9)	<0.001
Diameter of the largest foci in mm, mean ± SD	8.7 ± 5.9	9.4 ± 6.9	0.437
Number of removed lymph nodes within level VI, mean ± SD	13.8 ± 3.7	6.5 ± 2.7	<0.001
Number of macrometastatic lymph nodes, mean ± SD	6.4 ± 2.7	2.1 ± 1.0	<0.001

PTC was diagnosed in 130/548 (23.5 %) HT patients versus 643/8,597 (7.5 %) non-HT patients (*p* < 0.001)

*HT* Hashimoto thyroiditis, *PTC* papillary thyroid cancer

In the multivariable analysis, multifocal disease was identified as an independent risk factor for LNM (odds ratio, 3.99; 95 % confidence interval, 1.12 to 14.15; *p* = 0.033) (Table 3).

**Table 3:** Univariable analysis of factor associated with lymph node metastases in 130 patients with PTC and HT involved in this study

	No. of patients	Lymph nodes metastases (*n* = 94)	
		Odds ratio (95 % CI)	*P*
Sex			
M	20	0.10 (0.037–0.31)	<0.001
F	110	1.00	
Age (years)			
<45	52	0.29 (0.14–0.66)	0.003
≥45	78	1.00	
Smoker			
Yes	53	0.37 (0.16–0.81)	0.482
No	74	1.00	
Tumor size (mm)			
<10mm	80	3.02 (1.45–6.25)	0.003
≥10mm	50	1.00	
Multifocal disease			
Yes	28	3.99 (1.12–14.15)	0.033
No	102	1.00	
Extension through thyroid capsule			
Yes	16	0.44 (0.15–1.28)	0.133
No	114	1.00	

No significant differences were observed between the HT-PTC patients versus non-HT-PTC patients (Table 4) with respect to the prevalence of complications following thyroidectomy.

**Table 4:** Complications after thyroidectomy in 773 patients with PTC involved in this study in relation to the Hashimoto thyroiditis

	PTC in HT (*n* = 130)	PTC in Non-HT (*n* = 643)	*P*
Parathyroid found in pathological report, no. (%)	16 (12.3)	52 (8.2)	0.121
Hypoparathyroidism, no. (%)			
Total	48 (36.9)	216 (34.1)	0.465
Transient	45 (34.6)	203 (32.0)	0.498
Permanent	3 (2.3)	13 (2.0)	0.835
Unilateral RLN injury, no. (%)^a^			
Total	13 (5.0)	58 (4.5)	0.731
Transient	9 (3.5)	43 (3.3)	0.923
Permanent	4 (1.5)	15 (1.2)	0.619

*HT* Hashimoto thyroiditis, *PTC* papillary thyroid cancer, *RLN* recurrent laryngeal nerve

^a^RLN injury was calculated for nerves at risk and not for patients (there were 260 nerves at risk in PTC with HT group and 1,286 nerves at risk in PTC non-HT group)

## Discussion

More than 100 years after Hashimoto’s thyroiditis was first described, its etiology still remains not fully understood. Until early 50s of the last century, HT was a rarely described disease entity. The discovery and description of an unambiguous relationship between HT and papillary thyroid cancer by Dailey et al. in 1955 made numerous authors search for the causes of the phenomenon [23].

At present, according to numerous meta-analyses and data from the literature on the subject [1, 9, 24], papillary thyroid cancer coexists with Hashimoto’s thyroiditis approximately three times more often and the percentage of PTC in various reports is within a considerably sharp range of 0.5 % to above 58 %, with a peak at 76 % in a selected group of patients from Japanese and American populations (white American women). A visible increase in the incidence of the two diseases coexisting has been also observed within the past 20 years. This situation is undoubtedly affected by an increase in the incidence of autoimmune thyroid diseases, improvement of ultrasound techniques, higher availability of biopsy, or higher awareness of patients [1, 9, 13, 24–26].

Similarly as in the above quoted literature, in the presently analyzed material, in 23.5 % of the patients, papillary thyroid cancer coexisted with Hashimoto’s thyroiditis-type lesions. A threefold increase of the incidence of papillary thyroid cancer with the background of chronic autoimmune thyroiditis as compared to all patients with this cancer unambiguously points to an autoimmune background of the disease (HT-PTC: 130 = 23.5 % versus non-HT-PTC: 643 = 7.5 %).

The results presented in earlier and contemporary reports suggest that HT-PTC is significantly more frequently seen in the population of females in a lower age range, presenting with small lesions not exceeding 10 mm, multifocal disease, but also with statistically better prognoses as compared to the remaining group of patients. In the present study, women prevailed in both groups (*p* < 0.001), being aged slightly over 45 years, and the autoimmune background coexisted with multifocal form of papillary thyroid cancer with small primary foci (pT1a) [27–33].

To date, there has not been formulated a definite and unambiguous position on predisposition to development or inhibition of neoplastic process in patients with Hashimoto’s thyroiditis. Numerous publications on the subject only report final conclusions based on population studies [31, 32].

The presence of an autoimmune disease facilitates development of neoplastic lesions, but it is not entirely clear whether the disease per se predisposes to cancer development or whether it may provide a protective barrier against its spread as lymph node metastases or local recurrent disease [33].

At present, an important role in the pathogenesis of thyroid autoimmune diseases is played: FasL molecule and Fas receptor, inhibitor molecule like Bcl-2 protein, and the most common mutations in thyroid cancer include RET/PTC rearrangement, BRAF mutation, RAS mutation, or finally rearrangements leading to formation of PAX8-PPARg fusion oncogene. At present, the best known mutation form in papillary thyroid cancer is RET/PTC1 and RET/PTC3 sequence, also encountered in Hashimoto’s thyroiditis. Shel et al. detected RET/PTC1 mutation in as many as 95 % investigated patients, although they presented without clinical manifestation of lesions involving thyroid parenchyma. In view of the possible analysis of the BRAF gene originating from biopsy material (FNAB), reports have been published on employing the test to define high-risk groups.

We do not know which of the above-presented factors could have affected the number of metastases and the number of diagnosed cases of papillary thyroid cancer in the present material; nevertheless, we have noted both a high percentage (130 = 23.5 %) of papillary thyroid cancers (HT-PTC) as compared to (643 = 7.5 %) cancer patients in the non-HT-PTC individuals without the autoimmune disease and a statistically significant difference with respect to lymph node metastases to the central compartment of the neck (LNM: HT-PTC group: 94 of 130 (72.3 %) versus non-HT-PTC group: 164 of 643 (25.5 %)) [34–45].

Thus, molecular studies may become an important element of cytological diagnostic management, by the same token decreasing the number of dubious diagnoses. The considerable differences in biopsy results (0.4 %—Matesa et al. to 92 %—Singh et al.) may be a consequence of differences in defining lesions that require thyroid cytological diagnostic management in patients with Hashimoto’s thyroiditis [1, 46].

In the present material, the diagnosis of papillary thyroid cancer was confirmed in biopsy material only in 47 of 548 (8.04 %) HT patients. Although the number is very low, it is also confirmed in the literature on the subject. In all the remaining cases of limited nodular lesions situated in the thyroid parenchyma, specificity and sensitivity of the test was high, above 90 %. It is difficult to unequivocally state whether this occurs due to lymphocytic infiltrations, displacement of papillary cells by lymphocytes, or formation of reaction centers surrounding the primary focus. As it has been mentioned above, to date we do not know whether the presence of the afore-mentioned infiltrations is a factor that predisposes to tumor development or a reaction of the body to neoplastic disease. Diagnostic problems in evaluation of FNAB results continue to be stressed, both with respect to false-negative results and to overinterpretation of microscopic images with false-positive results [47–49].

A significant element in clinical assessment of papillary thyroid cancer is the size of a primary focus. As it has been mentioned, papillary thyroid cancer is generally characterized by a favorable prognosis. This is particularly true of patients with well-differentiated pT1a stage cancers, which do not exceed 10 mm in diameter. It should be noted, however, that even these forms show a fairly high capability to form metastatic foci. In the analyzed material, lymph node metastases to the central compartment of the neck were significantly more numerous in patients with cancer and Hashimoto’s thyroiditis. Ultrasonographic findings predominantly showed non-specific enlargement of cervical lymph nodes of the central compartment. The size of the primary focus in the majority of cases (80 = 61.7 %) did not exceed 10 mm (mean 8.7 ± 5.9 mm), and although the mean value was higher than the size reported by other authors, nevertheless, it provided a basis for diagnosing microcarcinomas [50, 51].

The multifocal form of papillary thyroid carcinoma in patients with Hashimoto’s disease was diagnosed in 28 (5.1 %) patients; in this group, 25 (89.3 %) metastases to the central compartment were observed. Thus, in light of data from the literature, one may assume that the size of the primary focus does not determine the presence or absence of lymph node metastases and coexistence of an autoimmune disease and papillary thyroid cancer does not significantly affect the number of foci but often coexists with their smaller size. Thus, Hashimoto’s thyroiditis may play a protective role, triggering improvement of therapeutic results and, by the same token, also improving the prognosis. Such data have been extensively reflected in the literature on the subject [33, 52–56].

Based on their observations of the two groups of patients, the present authors have concluded that coexistence of Hashimoto’s thyroiditis and papillary thyroid cancer is a more common phenomenon and affects almost one fourth of all patients. In the meta-analysis published in 2013, such a diagnosis accounts for only 1.2 % of biopsy diagnoses as compared to 27.6 % of ultimate results of histology following thyroidectomies. In turn, Shih et al. analyzed 474 surgical patients with coexisting HT and found papillary thyroid cancer in final histology in as many as 53 %, while “only” 28.05 % tumors were found in preoperative FNAB [57]. Thus, the remaining group consisted of patients without clinical and cytological features of neoplastic disease. Similar observations were made by Chinese authors—Lun et al. [33] emphasized differences in incidence of papillary cancer in patients with coexisting Hashimoto’s thyroiditis as compared to patients with preoperative diagnosis of nodular goiter (18.8 versus 7.2 %). The said authors also suggested that independent factors of cancer development included elevated serum TG antibody and TSH titers in patients with autoimmune thyroid disease [33].

And thus, may we passively observe patients with coexisting nodular lesions and Hashimoto’s thyroiditis? The question continues to be highly controversial [33, 49, 57].

Numerous authors are still inclined to employ markedly more conservative treatment of Hashimoto’s thyroiditis in keeping with the assumptions presented by Thomas et al. in 1981 in the *Annals of Surgery* that patients referred for surgical treatment of HT should meet the following criteria: the presence of a dominant mass with incomplete regression on suppressive therapy; progression of thyromegaly despite suppressive therapy; history or physical findings suggesting malignancy, e.g., irradiation, multiple endocrine adenomatosis syndrome, nerve paralysis, pain, tracheal compression, stipple calcification, and cervical lymph node enlargement; and the last: positive findings in FNAB. Such a position supports the view shared by some authors that treatment of Hashimoto’s thyroiditis is associated with a statistically higher probability of postoperative complications manifested as hypocalcemia and laryngeal nerves palsy [58, 59].

The above-presented various therapeutic strategies employed in patients with Hashimoto’s thyroiditis have led the present authors to presenting their own opinion. It is our belief that in view of the relatively high percentage of PTC in HT, the proposed strategy of surgical HT treatment should be total thyroidectomy. Prophylactic lymphadenectomy of compartment VI cervical lymph nodes remains to be considered, as it allows for a correct determination of the stage of a possible neoplastic disease and indications for further adjuvant therapy. Increasingly more often reports are published stating that primary total thyroidectomy not only allows for treating a disease already diagnosed based on the FNAB result but also contributes to decreasing the incidence of reoperations due to postoperative diagnosis of thyroid cancer. Analyzing the above-presented assumptions for treatment of Hashimoto’s thyroiditis with coexisting nodular lesions, we have to strongly emphasize that total thyroidectomy with resection of central compartment lymph nodes may be associated with damage to the recurrent laryngeal nerves and an inadvertent resection of inferior parathyroid glands. Nevertheless, we believe that the incidence of such complications in referral centers is low and comparable to operations performed due to indications other than autoimmune disease. In our material, we have not observed significant differences in the incidence of complications in patients with diagnosed papillary cancer with coexisting Hashimoto’s thyroiditis as compared to the remaining group [14, 18]. This point, extremely important in the context of the proposed radical procedures, is fully justified with respect to the presented in our material low diagnostic value of preoperative FNAB in nodular lesions and a simultaneous increase in incidence rates of cancer concomitant with Hashimoto’s thyroiditis in histology materials (the presence of PTC in HT being threefold higher as compared to the non-HT-PTC group).

It should be, however, stressed that the above assumptions are valid for referral centers, to which patients with HT should be sent. We believe that dissemination of this “radical thyroid surgery” strategy to low-volume thyroid surgery units should be preceded by extensive and well-designed training programs, followed by strict quality control assurance being now possible in some countries [18].

And thus, in our opinion, radical surgical treatment of patients with Hashimoto’s thyroiditis is an important prognostic factor in the strategy of therapeutic management that allows—in cases of diagnosing thyroid cancer—for initiating effective radioiodine adjuvant therapy, decreases the risk of local and regional recurrent disease, and facilitates postoperative patient monitoring through increasing sensitivity of postoperative TG determinations. In our view, the abovementioned considerations provide a good basis for taking into account radical surgical treatment based on the “cost–benefit” principles in cases with nodular lesions being present in an immunologically altered thyroid gland [14, 18, 19].
